# Ultrasonographic Features of Polycaprolactone Granulomatous Reactions: Case Series and a Literature Review

**DOI:** 10.1093/asjof/ojag116

**Published:** 2026-07-22

**Authors:** Shirin Habibi, Faezeh Khorasanizadeh, Ifa Etesami, Nasim Tootoonchi, Hamidreza Mahmoudi, Kamran Balighi, Ximena Wortsman

## Abstract

Polycaprolactone (PCL) dermal fillers are widely used to stimulate collagen production and achieve long-lasting aesthetic results. Despite a favorable safety profile, case reports have described adverse events, including granuloma formation. The authors present 4 cases of granulomatous reactions after PCL injections, characterized clinically by painless, firm subcutaneous nodules and ultrasonographically by hypoechoic lesions with surface echogenic foci and comet-tail artifacts. To raise awareness among aesthetic practitioners, the authors conducted a narrative review of the existing literature on PCL-associated granulomas. They discussed the role of ultrasound in supporting the diagnosis of granulomatous reactions following PCL injections. This review highlights the diagnostic utility of ultrasonography and summarizes reported cases to date, aiming to enhance recognition and management of this potential adverse event.

Level of Evidence: 4 (Therapeutic)

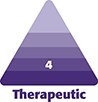

Polycaprolactone (PCL) is a popular filler known for its ability to stimulate collagen production and provide long-lasting aesthetic results.^1^ However, despite its favorable safety profile, there have been reports of adverse reactions following its use, including the formation of granulomas. Granulomatous reactions can occur because of foreign body responses to the filler, leading to significant patient discomfort and aesthetic concerns.^2^ High-frequency ultrasound is a diagnostic imaging technique that shows promise in aesthetic medicine for diagnosing complications.^2,3^ Granulomas typically exhibit ultrasound features, such as hypoechoic bands, tissue nodules, or pseudo-nodules with an oval shape and blurred, irregular outer edges surrounding the filler deposits. They may contain tiny hyperechoic punctate foci and occasionally produce calcifications and posterior shadowing. When compressed with the ultrasound probe, the shape of the tissue alterations remains unchanged.^4,5^

We reported 4 cases of granulomatous reactions after PCL injections referred to a tertiary center, presented with surface contour irregularities at injection sites and highlighted by their ultrasonographic features and individual management plans. In addition, we conducted a narrative review of previously reported PCL-associated granulomas using PubMed and Google Scholar with the keywords “polycaprolactone,” “PCL filler,” and “granuloma.” Articles published up to October 2025 were included based on their relevance to clinical presentation, imaging findings, and management. By enhancing understanding of these rare complications, we aim to contribute to the body of knowledge that guides safe and effective use of PCL in aesthetic treatments, with reduced reliance on biopsy when possible.

## CASE PRESENTATION

### Case 1

**Figure 1.**
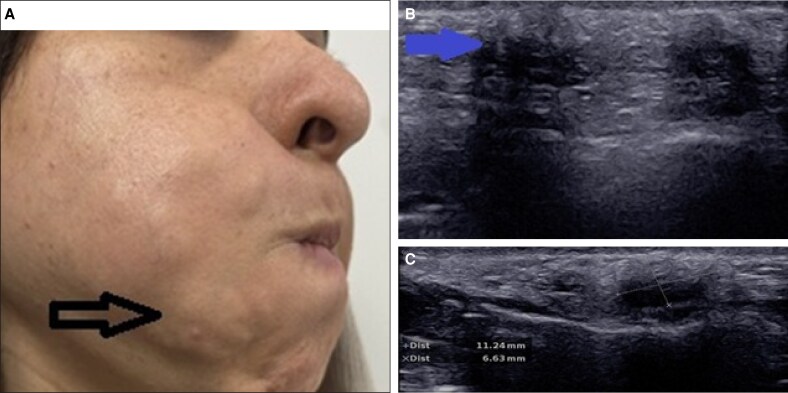
(A) A 36-year-old woman with a history of polycaprolactone (PCL) injection 2 years ago presents with multiple nonresolving nodular lesions in the right buccal region that developed 4 months after the injection. (B, C) Ultrasound images reveal multiple hypoechoic subcutaneous masses with echogenic foci on their surfaces (blue arrow in B), causing posterior shadowing, consistent with a PCL granulomas.

A 36-year-old female presented with firm, palpable nodules in the lower cheek following a PCL injection for cheek augmentation performed 2 years earlier by a dermatologist. Four months after the injection, she reported the development of these nodules at the injection sites, which remained stable despite receiving a hyaluronidase injection. She reported no history of underlying diseases, previous filler injections, or recent viral infections. Ultrasound imaging revealed multiple hypoechoic lesions in the superficial hypodermis, with echogenic foci observed at the surface, compatible with PCL granulomas (Figure 1). No other filler material was detected by ultrasound of the entire face. No biopsy had been performed regarding the ultrasound report. The patient was treated with oral prednisone at 1 mg/kg for 1 week, in addition to intralesional triamcinolone injection (20 mg/mL) in nodules, which resulted in significant improvement.

### Case 2

**Figure 2.**
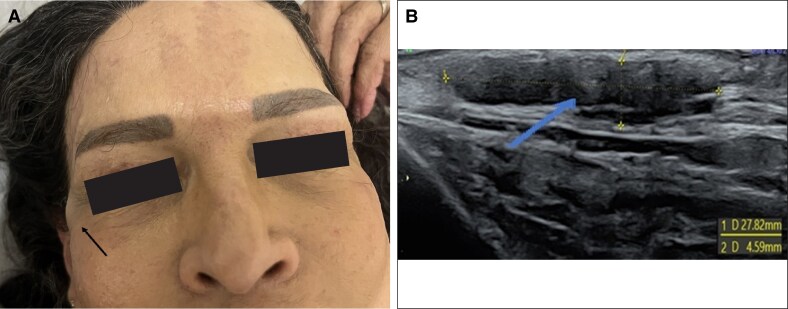
(A) A 69-year-old woman with a history of polycaprolactone (PCL) injection 1 year ago presents with multiple nodular lesions in the bilateral temporal region (black arrow), which developed 8 months after the procedure. (B) Ultrasound images show hypoechoic oval lesions with some echogenic foci (blue arrow) on their surfaces at bilateral temporal, suggesting PCL granulomas.

A 69-year-old female with a history of PCL injection in the bilateral temporal regions by a dermatologist 1 year ago presented with a recent sensation of lumps in the bilateral temporal areas. She did not mention any underlying disease, previous filler injection, or recent viral infections. Ultrasound imaging showed ill-defined hypoechoic lesions with hyperechoic spots at injection sites compatible with PCL granulomatous reaction (Figure 2). No additional filler material was detected on the ultrasound of the entire face. Patient refused biopsy and any treatment and was managed conservatively. She was advised not to inject any other filler until the resolution of the previous filler to prevent an adverse reaction.

### Case 3

**Figure 3.**
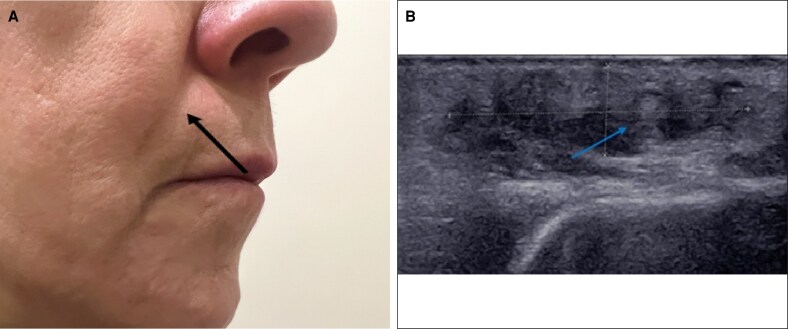
(A) Clinical and (B) ultrasound view of a 48-year-old female with a history of polycaprolactone injection in the bilateral nasolabial folds (black arrow in A). The ultrasound axial view shows a 32 × 9 mm ill-defined hypoechoic nodule with irregular borders and some echogenic foci compatible with granuloma (blue arrow in B).

A 48-year-old female presented with a history of PCL injection by a dermatologist in the bilateral nasolabial folds 10 months ago. She had a firm nodular sensation at injection sites for 2 months. The ultrasound showed a 32 × 9 mm ill-defined hypoechoic nodule with irregular borders and some echogenic foci compatible with granuloma (Figure 3). Ultrasound of the entire face did not detect any other filler material. Patient refused biopsy and any treatment and was managed conservatively. She was advised not to inject any other filler until the resolution of the previous filler to prevent an adverse reaction.

### Case 4

**Figure 4.**
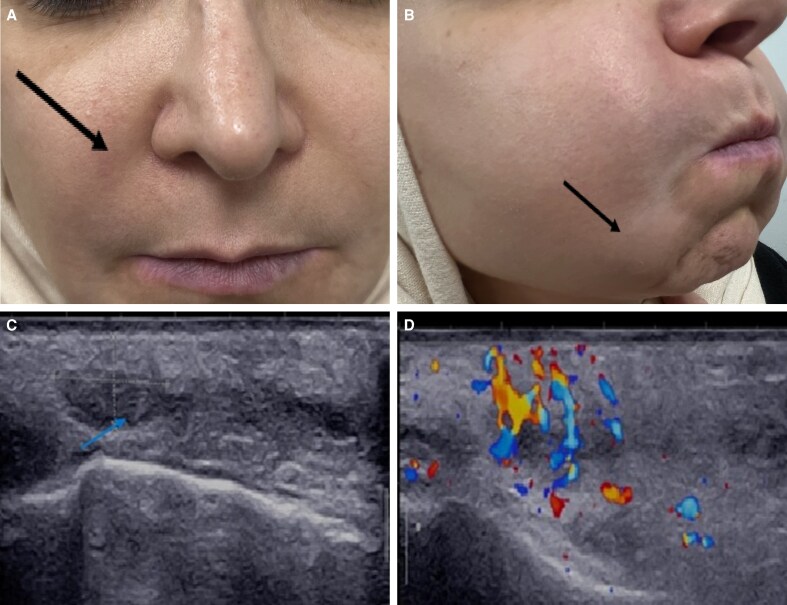
(A, B) A 52-year-old female with a history of PCL injection in the bilateral nasolabial folds and marionette lines (black arrows). (C, D) The ultrasound axial view reveals multiple hypoechoic nodules containing echogenic foci (blue arrows) and inner vascularity, which are compatible with granulomas.

A 52-year-old female reported PCL injection in the bilateral nasolabial folds and marionette lines by a dermatologist 2 years ago. She presented with a few firm nodules and sensations at the mentioned injected areas following a dental intervention 1 week ago. The ultrasound revealed multiple hypoechoic nodules containing echogenic foci and inner vascularity, compatible with granulomas (Figure 4). No other filler material was detected by ultrasound of the entire face. No biopsy had been performed. The patient was treated with oral prednisolone and doxycycline for 2 months, which resulted in significant improvement.

## DISCUSSION

PCL-based fillers have a favorable safety profile, a finding supported by daily clinical practice and postmarket surveillance worldwide over 10 years. The complication rate for PCL-based fillers has been reported as low. In a study of 780 patients treated with a PCL-based filler, there were no cases of intravascular injection, nodules, or granulomas during a 3-year observation period.^6^ Skrzypek et al reported the first case of a granulomatous reaction in 2018.^7^ Since then, a few publications have described this reaction. A foreign body granuloma associated with fillers is a form of chronic inflammation arising from a nonallergic reaction. It mainly consists of macrophages and multinucleated giant cells. These granulomas can develop several months to years after dermal filler use. Factors associated with an increased likelihood of granulomatous formation include nonbiodegradable fillers, larger injection volumes, previous infection or trauma at injection sites, and injection at the surface structure and chemistry of various dermal filler substances.^8-11^ PCL fillers are composed of 2 components: (1) carboxymethyl cellulose (CMC) and (2) PCL. Biologically, PCL is more closely linked to granuloma formation than CMC in fillers. PCL is a biodegradable polymer engineered to stimulate collagen production over time; however, its persistence within tissues may provoke foreign body reactions, potentially leading to granuloma formation—particularly after superficial injections, when large volumes are used, or in the presence of preexisting inflammation. Conversely, CMC acts as a carrier for PCL microspheres, is water-soluble, and is rapidly resorbed, making it less prone to induce a granulomatous response. Thus, the risk of granulomas with PCL fillers is largely attributed to the PCL component rather than the CMC.^2^ We identified only 10 case reports describing 13 patients with granuloma formation linked to PCL injection.^7,12-20^ Of these, 7 of 10 studies used ultrasound for diagnosis, and 7 included a biopsy to confirm granuloma. PCL fillers often appear as hypoechoic, irregular, or ill-defined lesions within the subcutaneous tissue, sometimes with posterior acoustic artifacts such as mini comet tails and perinodular hypervascularity. Treatment plans are heterogeneous; some cases improve with antibiotics (eg, doxycycline) or steroids (intralesional triamcinolone and prednisone), whereas others require escalation to disease-modifying therapies (eg, methotrexate) or combination approaches (including hyaluronidase or fluorouracil) and, in select instances, surgical excision or US-guided injections. Imaging features alongside clinical presentation guide management, with several reports noting partial or complete resolution only after multimodal treatment or when conservative medical therapy is insufficient. Overall, ultrasound is a useful diagnostic adjunct and can help monitor response to therapy in PCL filler–related granulomatous reactions (Supplemental Table 1). There is currently no literature detailing the specific ultrasound features of granulomas following hyaluronic acid or other filler materials compared with PCL injections. Diagnosis typically relies on the patient's history or pathology. However, the presence of echogenic foci with comet-tail artifacts on the surface of a hypoechoic nodule is more indicative of a PCL granuloma than a granulomatous reaction to other fillers. This distinction can help identify the underlying cause of the granuloma in legal cases, particularly when patients deny or forget the type of filler used.

We present 4 pictorial case reports from a tertiary center describing lump formation after filler injection. All 4 patients recalled Ellanse (PCL; Sinclair Pharma, London, United Kingdom) as the product used, and injections were performed by 4 different dermatologists. The injection sites reported in the cases are based on patient history and imaging findings rather than access to complete procedural records, and data on injection volumes and whether injections followed the manufacturer’s instructions were not available in the medical records. These limitations related to primary injections significantly constrain our ability to infer any causal link between the granulomas and the filler product. In particular, the absence of procedural data from the initial injections (eg, number of providers, technique, antisepsis, product batch, and injection settings) prevents definitive conclusions about whether factors at the time of injection contributed to granuloma formation. Furthermore, the absence of histopathological confirmation because of patient refusal, cosmetic concerns, and facial scarring limits diagnostic certainty.

Our study emphasizes the importance of awareness among aesthetic physicians regarding the potential for granulomatous reactions with PCL fillers, despite their overall favorable safety profile. Our findings also demonstrate the usefulness of ultrasound in identifying these adverse events, potentially reducing the need for biopsies, which can cause scarring and cosmetic issues. However, our findings are descriptive and observational, not causal, and future prospective studies with access to detailed injection histories (provider, technique, antisepsis, volume, and lot numbers) and histopathology would be required to assess causality and risk factors more reliably.

## CONCLUSIONS

Ultrasound can be used to support the diagnosis of granulomatous reactions following PCL injection.

## Supplementary Material

ojag116_Supplementary_Data
