# Supplementary material for: Ultrasonographic Features of Polycaprolactone Granulomatous Reactions: Case Series and a Literature Review
Source: Aesthet Surg J Open Forum. 2026 Jul 22;8:ojag116. doi: 10.1093/asjof/ojag116 (PMC13390709; doi:10.1093/asjof/ojag116)
Supplement: ojag116_Supplementary_Data [file ojag116_supplementary_data.docx]

|  | Author, year | Age and gender | Location | Clinical presentation | sonographic findings | Pathologic findings | Treatment |
| --- | --- | --- | --- | --- | --- | --- | --- |
| 1 | Moon et al./2017 | 36/male | Cheek, nasolabial folds, infraorbital | Several painless firm subcutaneous nodules | Ill-defined hypoechoic nodular infiltrative lesion | granulomatous inflammation with histiocytes and multinucleated giant cells | Doxycycline 100 mg twice daily for 1 month |
| 2 | Skrzypek et al./2019 | 68/female | Marionette line | small, firm, movable, non-tender nodules accompanied by a bluish discoloration. | A hypoechoic lesion with irregular borders, sized 0.378 cm × 0.416 cm, located within the subcutaneous tissue. 0.5 mm below the dermis | Numerous giant cells diffused on the matrix of fibrous connective tissue accompanied by macrophage and lymphocyte infiltration | No treatment |
| 3 | Philibert et al./2020 | 47/female | Cheeks & nasolabial folds | several nodules | No radiologic finding is mentioned | Confluent, well-circumscribed cellular nodules, surrounded by fibrosis with a few lymphocytic clusters, cellular and paucicellular zones, and numerous optically empty lacunae | 1x intralesional triamcinolone, hydroxychloroquine, and Isotretinoin (all ineffective). 10-20mg MTX^1^ per week for 12 months: effective |
| 4 | Chiang et al./2021 | 57/female | Tear trough | Asymptomatic yellowish nodule | No radiologic finding is mentioned | granulomatous inflammation, with epithelioid histiocytes, lymphocytes, and multinucleated giant cells in the dermis and subcutaneous fat | Excision |
| 5 | Ortiz-Álvarez et al./2021 | 74/female | Nasolabial folds and over both zygomatic arches | four firm, subcutaneous nodules, each measuring 2×2 cm | No radiologic finding is mentioned | chronic granulomatous inflammation with multinucleated giant cells involving the adipose tissue | 3 months of oral prednisone (0.7 mg/kg/day): ineffective  MTX (20 mg s.c. weekly) with prednisone (0.17 mg/kg/ day): effective |
| 6 | Ikonnikova,2021 | 36/female | nasolacrimal grooves | Painful symmetrical edemas of both nasolacrimal grooves and foci of brown pigmentation corresponding to their boundaries | Hypoechoic symmetrical heterogeneous formations of a rounded shape measuring 18 × 10 and 16 × 7 mm, moderate tissue swelling without a pathological increase in blood flow | fibro-fatty and granulation tissue fragments with pronounced lymphomacrophage infiltration and giant multinucleated foreign body cells, with scattered, uniformly round vacuoles. | Excision / lower blepharoplasty |
| 7 | Jun Ki Hong/  2022 | 51/female | both nasolabial  folds | Skin-colored painful firm nodule with perilesional contracture in the left perioral area | Poorly demarcated heterogenous hypoechoic lesions located within the subcutaneous layer, approximately 0.5 cm below the epidermis | Biopsy not performed | Oral antibiotics, HIFU^2^, Quantum Molecular Resonance Technology (5 sessions at 2-week intervals: effective |
| 8 | Shekarriz/ 2022 | 60/female | zygomatic arch and eminence, med. & lat. (SOOF)^3^, superficial malar fat pad, nasolabial fold bilaterally | facial deformity, malarial edema, and bilateral firm, tender nodules (70 × 50 mm and 60 × 40 mm). | Edema in the periorbital area and hyperechogenicity in suborbicularis oculi fat with a mass about 20 mm in length and multiple bright hyperechoic spots with mini-comet-tail artifacts in the hypoechoic matrix | Biopsy not performed. | Local injection of triamcinolone (1 mg/ cm2): ineffective  US-guided triamcinolone + 4 weeks minocycline: effective |
| 9 | Ianhez/ 2024 | 4 patients | medial limit of the temporal region | Not mentioned | poorly defined hypoechoic granulomatous tissue | Not mentioned | Not mentioned |
| 10 | Cerón Bohórquez,2024 | 46/female | gonial angle, pre-jowl sulcus, and chin | firm, nonpainful, non-fluctuant nodules accompanied by bluish skin discoloration. | Multiple hypoechoic nodules of varying size with posterior acoustic shadowing, irregular borders, bright hyperechoic spots, and “mini comet tail” artifacts along the borders, located in the subcutaneous fat and SMAS^4^ layer of the lower face  Color Doppler revealed perinodular hypervascularity. | nodular accumulations of histiocytic cells, multinucleated giant cells, and optically empty spaces | 4 intralesional steroid injections: ineffective  oral prednisolone and doxycycline for 2 months + three monthly ultrasound-guided treatment sessions:  Two sessions of triamcinolone and hyaluronidase, for the third session, fluorouracil was added: effective |

Supplementary Table 1. Reported cases of PCL-induced granuloma, MTX: methotrexate, HIFU: High-intensity focused ultrasound, SOOF: Suborbicularis oculi fat, SMAS: superficial musculoaponeurotic system
